# Standardised practices in the networked management of congenital hyperinsulinism: a UK national collaborative consensus

**DOI:** 10.3389/fendo.2023.1231043

**Published:** 2023-10-30

**Authors:** M. Guftar Shaikh, Angela K. Lucas-Herald, Antonia Dastamani, Maria Salomon Estebanez, Senthil Senniappan, Noina Abid, Sumera Ahmad, Sophie Alexander, Bindu Avatapalle, Neelam Awan, Hester Blair, Roisin Boyle, Alexander Chesover, Barbara Cochrane, Ross Craigie, Annaruby Cunjamalay, Sarah Dearman, Paolo De Coppi, Karen Erlandson-Parry, Sarah E. Flanagan, Clare Gilbert, Niamh Gilligan, Caroline Hall, Jayne Houghton, Ritika Kapoor, Helen McDevitt, Zainab Mohamed, Kate Morgan, Jacqueline Nicholson, Ana Nikiforovski, Elaine O'Shea, Pratik Shah, Kirsty Wilson, Chris Worth, Sarah Worthington, Indraneel Banerjee

**Affiliations:** ^1^ Department of Paediatric Endocrinology, Royal Hospital for Children, Glasgow, United Kingdom; ^2^ Department of Paediatric Endocrinology and Diabetes, Great Ormond Street Hospital for Children NHS Foundation Trust, London, United Kingdom; ^3^ Department of Paediatric Endocrinology, Royal Manchester Children's Hospital, Manchester, United Kingdom; ^4^ Department of Paediatric Endocrinology, Alder Hey Children’s Hospital, Liverpool, United Kingdom; ^5^ Department of Paediatric Endocrinology, Royal Belfast Hospital for Sick Children, Belfast, United Kingdom; ^6^ Department of Paediatric Endocrinology and Diabetes, University Hospital of Wales, Cardiff, United Kingdom; ^7^ Department of Dietetics, The Royal Infirmary of Edinburgh, Edinburgh, United Kingdom; ^8^ Department of Paediatric Surgery, Royal Manchester Children's Hospital, Manchester, United Kingdom; ^9^ The Children’s Hyperinsulinism Charity, Accrington, United Kingdom; ^10^ SNAPS, Great Ormond Street Hospital for Children NHS Foundation Trust, London, United Kingdom; ^11^ NIHR BRC UCL Institute of Child Health, London, United Kingdom; ^12^ Department of Clinical and Biomedical Science, University of Exeter, Exeter, United Kingdom; ^13^ Exeter Genomics Laboratory, Royal Devon University Healthcare NHS Foundation Trust, Exeter, United Kingdom; ^14^ Department of Paediatric Endocrinology, Faculty of Medicine and Life Sciences, King’s College London, King’s College Hospital NHS Foundation Trust, London, United Kingdom; ^15^ Department of Paediatric Endocrinology, Birmingham Children's Hospital, Birmingham, United Kingdom; ^16^ Paediatric Psychosocial Service, Royal Manchester Children’s Hospital, Manchester, United Kingdom; ^17^ Department of Paediatric Endocrinology, Barts Health NHS Trust, Royal London Children’s Hospital, London, United Kingdom

**Keywords:** congenital hyperinsulinism, glucose, hypoglycaemia, consensus, patient organisation, standardised practice, treatment

## Abstract

Congenital hyperinsulinism (CHI) is a condition characterised by severe and recurrent hypoglycaemia in infants and young children caused by inappropriate insulin over-secretion. CHI is of heterogeneous aetiology with a significant genetic component and is often unresponsive to standard medical therapy options. The treatment of CHI can be multifaceted and complex, requiring multidisciplinary input. It is important to manage hypoglycaemia in CHI promptly as the risk of long-term neurodisability arising from neuroglycopaenia is high. The UK CHI consensus on the practice and management of CHI was developed to optimise and harmonise clinical management of patients in centres specialising in CHI as well as in non-specialist centres engaged in collaborative, networked models of care. Using current best practice and a consensus approach, it provides guidance and practical advice in the domains of diagnosis, clinical assessment and treatment to mitigate hypoglycaemia risk and improve long term outcomes for health and well-being.

## Introduction

Congenital hyperinsulinism (CHI) is a rare condition characteristed by severe and often refractory hypoglycaemia in infants and young children, due to inappropriate excess insulin secretion from pancreatic β-cells (1). CHI has heterogeneous aetiology, but the majority are due to different underlying genetic variants (2) most commonly in the ATP sensitive K^+^ channels coupling glucose levels and insulin secretion.

Estimates of prevalence of CHI vary from 1 in 50, 000 to 1 in 2, 500, the latter in populations with high consanguinity rates (3–6). In the UK, the minimal incidence of CHI measured by genetic testing referral rates for hyperinsulinism persisting beyond 6 months of age, is 1 in 28, 389 live births (7). CHI represents a significant clinical burden, with an estimated annual cost of illness to the UK National Health Service of approximately £3, 408, 398 (8), not counting long-term costs from neurodisability.

CHI commonly presents in the neonatal period, with initial investigations and management initiated by general paediatric or neonatal teams. While a number of published reviews have summarised broad management principles and strategies and an international consensus guideline is due for publication, there is no clinical practice collaborative consensus that underpins the shared network treatment of CHI (9–11). This group has aimed to collaborate and harmonise current best practice to produce a working guideline for the management of CHI in the UK, with a focus on practical aspects including diet, medication changes and monitoring. This guideline is intended for use by general paediatricians, neonatologists, paediatric endocrinologists, surgeons, specialist nurses, dietitians and speech and language therapists, as well as GPs, involved in the day-to-day management of CHI patients through an effective network of care.

## Methods

A consensus group was derived from healthcare professionals with extensive experience in the management of CHI in centres across the UK (comprising Northern Ireland, Wales, Scotland and England) forming the CHI Special Interest Group (SIG), a subcommittee of the British Society for Paediatric Endocrinology and Diabetes (BSPED). The group also included patient representatives from the UK CHI Charity, Children’s Hyperinsulinism Charity (CHC). Members of the group were tasked with writing components for discussion. The group met virtually on 8 occasions over a period of 18 months at regular intervals (ending March 2023) to discuss available evidence and experience, with additional separate smaller group meetings. The highest level of evidence from the current scientific literature was used as the basis of recommendations agreed by the entire group. The GRADE criteria was not adopted by the group on grounds of paucity of high quality evidence in formulating practice advice in a networked model and the concomitant development of an international CHI guidance providing general recommendations. Where evidence was deemed insufficient or inadequate to be able to make a definitive recommendation, the authors discussed a consensus of centre experiences to frame a working recommendation appropriate for the network. Consensus was achieved by presentation of evidence in published literature, followed by discussion among group members and subsequent voting to determine a significant majority (>80%).

The terminology to describe hypoglycaemia from hyperinsulinism is variable and can include Congenital Hyperinsulinism (CHI), Hyperinsulinaemic Hypoglycaemia (HH), Hyperinsulinism (HI). For the purposes of this guideline, CHI will be adopted as the preferred term regardless of genetic aetiology or the age of onset of illness.

The specific areas in CHI, covered by the consensus are:

• Presentation and diagnosis;
• Management options including fluids, feeds, medications and surgery;
• Considerations for discharge from hospital;
• The role of the extended multidisciplinary team (MDT);
• Long-term management and outcomes;
• Potential future therapies.

### Presentation and Diagnosis

#### Hypoglycaemia: diagnostic and therapeutic thresholds

Hypoglycaemia occurs when blood glucose is low, ie less than the normal range (4.0 and 6.0 mmol/L) (12). As patients with CHI have greater risk of neuronal injury and neurodevelopmental sequelae due to severe hypoglycaemia and the absence of ketone production, it is pragmatic to keep glucose levels close to the normal cut-off of 4.0 mmol/L as possible rather than risk possible neuronal injury with lower levels (9).

The definition of neonatal hypoglycaemia itself is controversial. Studies suggest using a cut off glucose of 2.0 mmol/L in asymptomatic neonates without risk factors for CHI, such as fetal distress and intra-uterine growth restriction, in contrast to more traditional levels of 2.6 mmol/L (13). However, there is some evidence of the risk of long-term executive motor function and visual motor function impairment at levels of both 2.0 and 2.6 mmol/L, albeit without correlation to school performance (14, 15), implying the absence of an agreed safe cut off glucose level. In view of the uncertainties over an agreed hypoglycaemia threshold, it is prudent to aim for a glucose level at or near the normal level.

#### Glucose threshold for investigation – 3.0 mmol/L

For operational purposes, it is important to define a cut off glucose level to commence (16) investigations and treatment for hypoglycaemia. An international consensus defined this as 3.3 mmol/L (17); however, this level may be too high and could lead to over-investigation and the finding of detectable insulin levels in many neonates without CHI, with consequent over diagnosis of CHI. According to the experience of the specialist centres a level of 3.0 mmol/L is a reasonable compromise between 3.3 mmol/L and the lower traditional threshold of 2.6 mmol/L and could be used as a practical threshold, particularly in the presence of severe hypoglycaemia associated with a high glucose infusion rate (GIR).

The timing of hypoglycaemia screening is complicated by transitional hypoglycaemia in the first 48-72 hours of life when neonates adapt to extra-uterine conditions. Guidelines have been developed by the British Association for Perinatal Medicine (BAPM) for the management of neonatal hypoglycaemia, although it is important to remember these are for generic neonatal hypoglycaemia and not specifically for CHI 18). Current international recommendations suggest investigation for CHI beyond this period (17). However, severe and/or recurrent hypoglycaemia, undetectable blood glucose levels at any time, or two or more episodes <3.0 mmol/L with glucose infusion rate (GIR) >8 mg/kg/min within the first 48 hours of life, should alert the clinician to the potential diagnosis of CHI even within this period. A high GIR beyond 48 hours after birth in the context of severe and recurrent hypoglycaemia is a a strong pointer to CHI over transitional hypoglycamia or metabolic causes of hypoglycaemia (19).

GIR, a practical measure of glucose requirement, can be calculated from the rate of infusion, concentration of dextrose and body weight [mg/kg/min) = (Fluid rate (ml/hr) X % Dextrose)/(Weight (kg) X 6)] or by using online calculators (20, 21). A normal GIR in the neonatal period is 4-6 mg/kg/min; rates exceeding 8 mg/kg/min suggest the diagnosis of CHI (9, 17).

The biochemical diagnosis of hyperinsulinism relies on the finding of a detectable insulin level at hypoglycaemia. Insulin has a short half life of around 6 minutes (9) and therefore may be missed if hypoglycaemia blood screening samples are delayed. If insulin is undetectable but clinical features point to CHI, further diagnostic samples may be required. Excess insulin secretion suppresses ketones in addition to producing hypoglycaemia and may be used as a biochemical surrogate for hyperinsulinism. Therefore, the combination of hypoglycaemia (at or below 3.0 mmol/L), suppressed ketones and fatty acids as well as an increased GIR is diagnostic of CHI.

In the absence of measurable insulin levels during hypoglycaemia or a high GIR, an alternative cause should be pursued in discussion with the regional endocrine and/or designated CHI specialist centre. The possibility of an underlying metabolic disorder should also be considered, particularly if serum ammonia levels are raised or if there is lactic acidosis. Hypopituitarism and adrenal insufficiency may also need to be excluded as causes of hypoglycaemia.

#### Glucose threshold for treatment – 3.5 mmol/L

It is aspirational to aim for glucose > 4.0 mmol/L in the treatment of hypoglycaemia due to CHI; however, this may require significant escalation of treatment (for example greater concentrations of dextrose) that may lead to therapeutic side effects. The risk of overtreatment has to be balanced against the risk of neurological injury at lower glucose levels (9); by consensus a practical therapeutic glucose threshold level of ≥3.5 mmol/L was agreed.

#### Initial investigations

A “hypo screen sample” should be obtained at the time of spontaneous hypoglycaemia and before treatment with glucose (**Table 1**). Samples obtained after glucose treatment may misrepresent insulin, free fatty acids (FFA) and beta hydroxybutyrate (BOHB) levels at the time of hypoglycaemia, thereby delaying the diagnosis of CHI. If insulin samples cannot be obtained at the time of hypoglycaemia and the diagnosis of CHI is doubtful, hypoglycaemia may need to be induced with careful glucose monitoring to reach a diagnosis after discussion with the regional endocrine/CHI centre. Pre-prepared “hypopacks” which contain blood sample containers (**Table 1**) can be helpful. In cases of difficult sample collection or small blood volumes, priority should be given to plasma glucose and serum insulin for analysis. Plasma glucose samples should always be taken peripherally to avoid any contamination from intravenous glucose that could cause false interpretation of results. It is important to ensure that insulin samples are analysed with a rapid turnaround time (expected 2-3 working days) to avoid delays in establishing the diagnosis of CHI. Most modern assays now detect insulin at low concentrations. However, with highly sensitive insulin assays (22), there is potential for overdiagnosis of CHI. As laboratory analyses vary, assay-dependent insulin cut-off levels (and other analytes such as BOHB and FFA) should be determined for clinical significance; in most cases insulin levels above 12 pmol/L (2 mU/L) indicate meaningful hyperinsulinism. In case of doubt about the diagnosis of CHI, a discussion with the regional endocrine and/or CHI centre is advised.

**Table 1:** Initial hypo screen investigations (those in bold indicate a higher priority).

Samples taken at the time of hypoglycaemia	May be taken after hypoglycaemia correction	First urine after hypoglycaemia
- **Glucose,** lactate - **Insulin, C-peptide** - FFA, BOHB - Cortisol * - GH *	- Acylcarnitine - Plasma amino acids - Ammonia - Electrolytes - Liver function	- Urine organic acids

FFA, free fatty acids; BOHB, beta hydroxy butyrate; GH, growth hormone.

To check specimen/tube/volumes with local laboratory.

*GH and cortisol axis may need to be evaluated to exclude hypopituitarism.

The level of insulin at the time of hypoglycaemia does not correlate with the severity of the disease. Insulin may be undetectable in up to 20% of cases (23). Therefore in the presence of clinical features of CHI, the diagnosis may hinge on the evidence of insulin action, i.e., low ketones/BOHB and low free FFA. Point of care testing for BOHB can be useful to demonstrate low ketone production, particularly in older children. In neonates on dextrose infusions or frequent feeds, BOHB is less reliable but should be used if facilities are available. However, the presence of modest ketones is not sufficient to exclude CHI (22).

C-peptide can also aid the diagnosis, particularly where hyperinsulinism is suspected but not proven. A level ≥ 0.5 ng/mL (>165.5 pmol/L) may offer a high sensitivity of a CHI diagnosis (23). However, as C-peptide has a longer life than insulin, it may represent secretion prior to hypoglycaemia. Further, lower levels of detection are likely to be assay dependent; therefore, C-peptide cut-offs to indicate high probability of CHI may vary between centres.

The possibility of exogenous insulin administration as a cause for hypoglycaemia should be considered if C-peptide levels are undetectable but insulin is present at hypoglycaemia. If factitious and fabricated illness (FII) is suspected, samples should be obtained specifically for insulin analogues. It is important to highlight that not all insulin assays are able to detect insulin analogues and some assays only detect certain insulin analogues, but not others. If the diagnosis of CHI is in doubt or if FII is considered by the clinical team, the regional endocrine and/or CHI centre should be contacted.

#### UK referral pathways and CHI specialist centres

NHS England commissions a Highly Specialised Congenital Hyperinsulinism (CHI) service in England. CHI patients in Wales and Northern Ireland can also be referred to these centres through separate arrangements. The regional Scottish endocrine centres manage patients with CHI locally in Scotland in collaboration with the English CHI centres.

The CHI centres have established advice and referral networks which include tertiary paediatric endocrinology centres and district general hospitals (DGH). It is expected that the diagnosis and early treatment of hypoglycaemia in neonates and infants is considered in local units before proceeding with a referral to a CHI centre. If CHI is unlikely, the patient should be managed locally. In cases of uncertainty of diagnosis, the networked CHI centre should be consulted. If the cause of hypoglycaemia is likely to be of metabolic aetiology, the regional centre for inherited errors of metabolism should be contacted. In some cases, it is not clear if hypoglycaemia is due to CHI or metabolic aetiology; in such cases both CHI and metabolic opinions should be sought.

**Figure 1:**
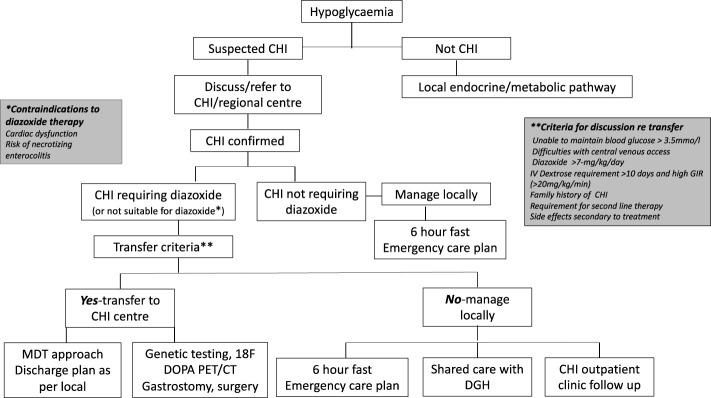
Referral pathways and criteria for congenital hyperinsulinism diagnosis and treatment in a networked model of care. CHI, congenital hyperinsulinism; DGH, district general hospital.

The model referral pathway for CHI is shown in **Figure 1**. The regional endocrine centre should consider liaision with the CHI centre soon after diagnosis of CHI to discuss investigation and treatment options and if referral to a CHI centre might be required.

The CHI centre should communicate effectively with the local hospital to discuss efficacy of treatment, safety issues and follow up plans in a network care plan. If a patient responds to diazoxide in a dose less than 7 mg/kg/day without side effects, as per consensus, care could be retained locally, with update on progress at regular intervals until hospital discharge. If the dose of diazoxide is increased to or beyond 7 mg/kg/day due to insufficient response, unacceptable side effects, or persistent requirement for intravenous dextrose, the local hospital should discuss with the CHI centre for potential transfer. The CHI centre should assume responsibility for the decision to transfer based on individual circumstances. If a CHI patient shows signs of resistance to diazoxide, second line treatment with a synthetic somatostatin analogue such as octreotide may be required. In most cases, octreotide should be commenced and managed in a CHI centre. Additionally, the CHI centre should be involved if surgical central venous access or pancreatic surgery is envisaged. It is accepted that the model (DGH-Regional Endocrine Centre-CHI centre) may not apply to all hospital centres depending on local expertise, preferences and arrangements and may require local adaptation for patient need. Nonetheless, the principles of efficient networked care remain the same, allowing for variations in the model and potential applications in countries outside the UK.

#### Acute and immediate management

The immediate treatment goal in CHI is to achieve a safe glucose level to prevent the risk of hypoglycaemia-induced brain injury. Hypoglycaemia should be treated promptly, once hypoglycaemia screening bloods have been taken. A bolus of 2.5mls/kg of 10% glucose is recommended (24) to treat acute hypoglycaemia followed by a continuous glucose infusion to prevent rebound hypoglycaemia. Subsequent smaller bolus doses of 1-2mls/kg of 10% glucose (if required) are suggested in CHI patients to avoid rebound hypoglycaemia.

In most cases, oral or nasogastric feeds, even with increased carbohydrate content, are not enough to attain normoglycaemia and intravenous access is required. As GIR is high in CHI, failure to correct hypoglycaemia with 10% glucose at a standard maintenance rate, makes CHI likely. A 10% glucose infusion of 5 mL/kg/hr (or 120 mL/kg/day) equates to a GIR of 8.3mg/kg/min and if hypoglycaemia persists with this GIR, the possibility of hyperinsulinism should be investigated. Until biochemical diagnosis of hyperinsulinism is confirmed, initial glycaemic stabilisation should be achieved with a high concentration glucose infusion (15-20%) administered by a central venous catheter. In addition, glucagon administration by intravenous/subcutaneous infusion is useful for initial glucose stabilisation, reduction of the total fluid requirement and a reduction in the potential risk of fluid overload associated with diazoxide treatment (25). At present, continuous subcutaneous glucagon infusions are not available for home use, due to instability of glucagon susceptible to fibrillation and precipitation in aqueous solutions. Soluble glucagon analogues have been successfully investigated in clinical trials and may become available for use in the near future (26).

**Figure 2:**
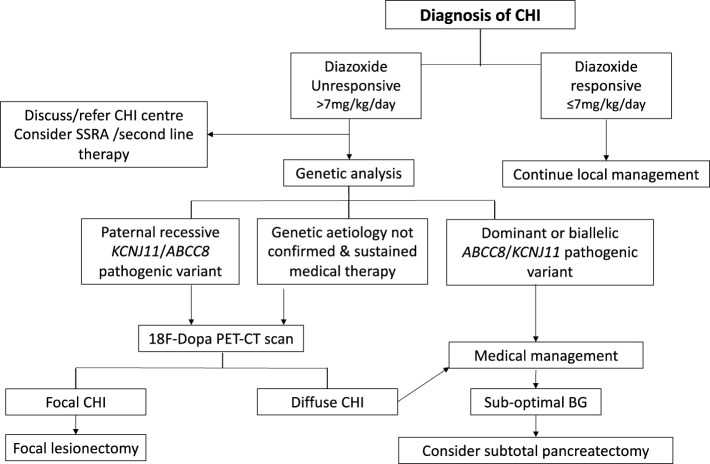
Clinical pathway of diagnosis and management for patients with congenital hyperinsulinism (CHI). Clinical decisions hinge on criteria such as diazoxide responsiveness, genetic investigations, 18 Fluoro Dopa PET-CT/MR imaging and response to medical therapy.

Following initial glycaemic stabilisation, definitive treatment of CHI should be considered with a view to safe discharge from the hospital. Treatment with diazoxide is the first line of definitive clinical management and is pivotal to a successive clinical decision pathway outlined in **Figure 2**. Diazoxide responsiveness is a key criteria that determines requirement for CHI specialist care, genetic investigations and second line therapy with octreotide. An escalating diazoxide dose > 7 mg/kg/day (indicating partial response) (9), consolidated by consensus, was deemed unsuitable for local care and more appropriate for CHI specialist care. Patients requiring higher doses of diazoxide or those unresponsive to diazoxide have a higher propensity for monogenic aetiology (9) requiring genetic investigations utilising a targeted gene panel to screen for disease-causing variants. In patients with early indication of failure of medical therapies and high GIR, rapid analysis of the *ABCC8* and *KCNJ11* genes is required to expedite the potential diagnosis of focal CHI. Discussion with the local CHI centre regarding the urgency and type of genetic testing is advised, especially if there is a family history of diabetes and large birth weight infants.

### Medication

#### Glucagon

Glucagon increases endogenous glucose production by stimulating glycogenolysis, gluconeogenesis and lipolysis, which is typically inhibited in the presence of insulin excess. It can be used as an intramuscular injection in an emergency or as a continuous intravenous or subcutaneous infusion. The starting dose of glucagon is 2.5-5.0 micrograms/kg/hour, with dose escalation in steps of 2.5 mcg/kg/hr to maintain glucose levels above 3.5 mmol/L (**Table 2**). The maximum dose of glucagon generally used is 20 mcg/kg/hr (25, 27). To prepare a continuous infusion, glucagon formulation for intramuscular injection can be diluted in 0.9% sodium chloride or 5% dextrose. Glucagon solution should be changed every 12-24 hours to avoid catheter occlusions due to glucagon instability and precipitation in prepared solutions (28). It is preferable not to use line filters to minimise the risk of line occlusion. Glucagon can be administered peripherally or via central catheters with clear fluids, but avoided with parenteral nutrition or high concentration of dextrose (>15%), as the solute load might aggravate precipitation. The use of additional clear fluids down the same line as glucagon infusion can help to reduce precipitation and improve delivery of the active drug.

**Table 2:** Drug dosing and monitoring.

Drug	Dose	Pre initiation	Post initiation	Monitoring	Side effects/risks
Diazoxide	Initial 2-5mg/kg/d in 2-3 divided doses Increased to 20mg/kg/d	Fluid restriction 130-150 ml/kg/d- for at least 24-48 hrs prior to starting ECHO	ECHO- 10-14 days later*	UEs, urate, FBC,- 4-6 monthly	Fluid retention, Pulmonary hypertension, NEC, neutropenia, thrombocytopenia, hypertrichosis
Chlorothiazide	Initial 7mg/kg/d in 2 divided doses			UEs	Hyponatremia
Octreotide*	5-30mcg/kg/day, usually given 6-8hrly by sc injection	Abdo US, LFTs		LFTs, TFTs Growth, Abdo US- annually	NEC, biliary sludge, growth faltering, GI disturbance, cholelithiasis, pituitary hormone suppression
Glucagon (1000mcg/ml)	5-20mcg/kg/hr (start at 2.5-5.0 mcg/kg/hr)			Infusion set to be changed every 12 hours	IV line occlusion, NME

* especially if clinical concerns/existing cardiac issues. FBC, full blood count; GI, gastrointestinal; IV, intravenous; LFTs, liver function tests; NEC, necrotizing enterolitis; NME, necrolytic migratory erythema; TFTs, thyroid function tests; sc, subcutaneous; UEs, urea and electrolytes; US, ultrasound.

Glucagon is helpful to reduce the fluid requirement in neonates predisposed to fluid overload and in those at risk of developing pulmonary hypertension following treatment with diazoxide (29). From a glycaemic perspective, glucagon treatment is safe and effective in reducing GIR (25, 27). Glucagon associated side effects are infrequent; however it is important to recognise necrolytic migratory erythema (NME), a rare but severe form of rash associated with high doses over prolonged periods and disappearing after discontinuation (9). Prolonged glucagon may also cause vomiting, weight loss and coagulopathy. For the latter, particularly in those requiring high concentration (≥15%) glucose, prophylactic anticoagulation with enoxaparin should be considered to avoid central venous catheter-related thrombosis (30).

#### Diazoxide

Following an established diagnosis of CHI, treatment with oral diazoxide should be started as first line treatment if there is no spontaneous resolution of hypoglycaemia. Diazoxide, an oral nondiuretic benzothiadiazine, acts on the sulphonylurea receptor sub-unit of the ATP-sensitive potassium (KATP) channel to keep the channel in the open conformation leading to β-cell membrane stabilisation. This in turn, causes a reduction in calcium influx into the β-cell thereby reducing calcium mediated insulin efflux. Whilst diazoxide is effective in most individuals with non-KATP channel CHI, the majority of patients with a KATP channel mutation show poor response to the drug, although those with mild dominantly acting KATP channel mutations may respond well to diazoxide. There are also rare reports of infants with biallelic pathogenic variants in *ABCC8* being diazoxide responsive and a few patients with known pathogenic variants in *ABCC8* may have a partial response (31–33) although satisfactory glycaemic stability is not always achieved.

Diazoxide related side effects have been described in observational studies (34) with 10% patients expected to show minor or major concerns. In the early stages of use, diazoxide therapy often exacerbates fluid retention, necessitating the use of a diuretic such as chlorothiazide. Pulmonary hypertension can occur in 2-7% patients, following diazoxide therapy, arguably more so in those with pre-existing cardiovascular abnormalities. Therefore, an echocardiogram (ECHO) to assess cardiac structure and pulmonary artery pressures before diazoxide is recommended. A repeat ECHO can be considered 10-14 days after starting diazoxide treatment, especially if there are clinical concerns (29, 34–36). In cases where an ECHO cannot be easily obtained, close monitoring for cardiovascular compromise should be ensured until a cardiac review is available. In infants with previous but resolved pulmonary hypertension, diazoxide should be used with caution.

Treatment with diazoxide mandates a fluid volume restriction, including feeds and intravenous infusions to total of 130-150ml/kg/day in term neonates for 24-48 hours prior to diazoxide therapy to reduce the risk of pulmonary hypertension. In premature babies with higher risk factors for pulmonary hypertension, fluid volume may need to be restricted further to 120ml/kg/day. Volume reduction may be achieved by high glucose concentrations delivered by central venous catheters and/or by the use of glucagon to reduce glucose dependence.

Following diazoxide use, fluid volumes may be liberalised gradually in the absence of pulmonary hypertension, although a total fluid volume of >150ml/kg/d should be generally avoided in the first 2 weeks of life.

A starting diazoxide dose of 2.0-5.0 mg/kg/day in 2-3 divided doses is suggested by consensus, together with a chlorthiazide dose of 7mg/kg/day in 2 divided doses (**Table 2**). The lower dose of diazoxide may be considered appropriate in neonates likely to be diazoxide responsive and sensitive, for instance in those where the hypoglcaemia is due to perinatal stress induced hyperinsulinism, including intra uterine growth restriction and being small for gestational age (37, 38).

If pulmonary hypertension is suspected, diazoxide therapy should be discontinued immediately, as dose reduction is not effective in mitigation. Instead, further fluid restriction may be required with the need for high concentration glucose and/or glucagon to reduce the risk of hypoglycaemia. Additionally, an urgent cardiac opinion should be sought for confirmation of pulmonary hypertension.

Diazoxide use can be associated with other problems, including necrotising enterocolitis (NEC) (39, 40), more so in those with predispositions such as prematurity (40). Other side effects include neutropenia and thrombocytopenia in upto 9% of patients (10, 36) prompting the need to check blood counts both at initiation and in follow up. If neutropenia and/or thrombocytopaenia are present prior to treatment, diazoxide should be used with caution with regular monitoring. Rarer side effects include pericardial effusion requiring surgical drainage and paradoxical hypoglycaemia with high dosage (41, 42). In the long term, it is common to observe excess hair growth with doses > 5mg/kg/day (36). **Table 1** considers the dosage, monitoring guide and side effects of diazoxide and other drugs in CHI.

In view of the proclivity to side effects, it is advisable to start diazoxide in a lower dose, escalating upwards to a maximum of 20mg/kg/day, depending on the response. A dose in excess of 15 mg/kg/day is rarely effective and indicates diazoxide unresponsiveness. The majority of patients with biallelic pathogenic variants in *ABCC8* and *KCNJ11* are diazoxide unresponsive, necessitating second line treatment. In such cases, diazoxide can be stopped abruptly without gradual de-escalation. Further, it is advisable to avoid combination therapies in CHI to reduce the risk of therapy related major side effects.

Following a satisfactory glyaemic response to diazoxide, an age appropriate fast should be undertaken, prior to discharge from the hospital (discussed in a following section).

In the longer term, monitoring of growth, renal function, urate/uric acid and blood count is recommended. It is not necessary to increase the dose of diazoxide progressively with gain in weight, if glucose levels remain stable. In such cases the therapeutic strategy would be to “out grow the dose”, allowing for a gradual reduction in the dose for body weight. In those patients on previously higher doses of diazoxide (>5mg/kg/day), with relative dose reduction to <2-3mg/kg/day, as agreed by consensus, active reduction could be considered. Diazoxide dose reduction can be managed safely at home under supervision by the treatment team. These could include frequent virtual consultations by specialist nurses with prescribing authorisation. However, if stopping or reducing diazoxide therapy poses potential risks, inpatient admission for monitoring may be required. For those successful in active home withdrawal of diazoxide, a safety fast (age appropriate) and hospital admission is generally recommended; this can be done following discussion with the clinical team.

In all cases, initiation of diazoxide treatment should be accompanied by diuretic use. However, fluid retention becomes less problematic beyond the age of one year and chlorthiazide can be safely discontinued, especially if the diazoxide dose remains small (< 5 mg/kg/day) and does not require escalation.

#### Octreotide

Synthetic Somatostatin Analogues (SSA) such as Octreotide are used as second line therapy for children with CHI who are not responsive to diazoxide (43) (**Figure 2**). SSAs act through somatostatin receptors to inhibit cAMP mediated insulin secretion. Octreotide, a short acting SSA, is administered either as 6–8 hourly subcutaneous injections or by continuous intravenous or subcutaneous infusion.

The starting dose of octreotide is usually 5 micrograms/kg/day,which should only be initiated in a CHI specialist centre or regional centre with expertise/experience (**Table 2**). Initial administration of octreotide often leads to hyperglycaemia, but the effect is not sustained. Loss of initial effect or tachyphylaxis sets in after 48 hours when dose increments are required to prevent hypoglycaemia. The dose of octreotide can be increased in 2.5 microgram/kg/day steps to a maximum of 30 microgram/kg/day. Beyond this dose, side effects become apparent, with only marginal improvement in efficacy.

Octreotide may be administered via a continuous subcutaneous infusion using an insulin pump (44). The responsibility for initiating and advising on continuous octreotide infusions lies with the CHI centre with special consideration given to variable drug concentrations, ensuring correct conversion of micrograms to insulin units and anticipation of pump malfunction.

Octreotide acts on many tissues and has an impact on the hepatobiliary system with reports of hepatitis, biliary sludging and gall stones (43). Infants treated with octreotide have been reported to develop NEC, probably as a result of reduced splanchnic blood flow (45–47). Octreotide potentially reduces pituitary growth hormone production; while short stature is not commonly recognised, this may also be under-reported (48).

An alternative to short acting octreotide is the use of long acting SSA like octreotide LAR (e.g. Sandostatin LAR™ or Olatuton™) and somatuline autogel (e.g. Lanreotide™) (49–52). These can be considered in infants over 6 months of age and offer the prospect of once monthly subcutaneous or intramuscular injections, thereby reducing the need for multiple daily injections or cumbersome pump therapy. The efficacy profile seems satisfactory although studies remain observational without a control arm to judge true benefit over harm. Further, the use of long acting SSA is complicated by similar side effects as octreotide with the additional disadavantage of the persistence of side effects due to long interval dosing.

Alternative SSAs such as pasireotide have been used in difficult cases but efficacy remains contentious (53). It is not recommended to use pasireotide or other repurposed medications unless within a research capacity.

#### Feeds

Management of feeds is integral to the clinical management strategy in patients with CHI. Infants may require carbohydrate supplements alongside medications to facilitate weaning from intravenous dextrose to enteral feeds. Additional carbohydrates can be provided in the form of glucose polymers, high energy formula, pre-term formulas or breast milk fortifiers. It is recommended that infants requiring additional oral carbohydrate to prevent hypoglycaemia should be referred to an experienced paediatric or neonatal dietitian.

Addition of a glucose polymer should be started in a low dose at 1 - 2.5% (**Appendix 1**). An increase can be considered after 48 hours if no signs of intolerance are noted, such as vomiting or loose stools. Further increments can be made every 48 hours by 1 – 2%, up to a maximum of 5%, i.e. not exceeding 12.5% total carbohydrate including the carbohydrate content already provided in milk feeds. Examples of some infant milks and supplements for infants with CHI are shown in **Appendix 1**.

In neonates at increased risk of developing gut ischaemia such as **NEC**, extreme caution should be exercised in the use of supplemental carbohydrates that increase solute osmolar load. Only following discussion and assessment by an experienced paediatric/neonatal dietitian should a glucose polymer be added in such cases.

Ongoing dietetic supervision is required as the protein to energy ratio often becomes distorted with carbohydrates supplementation, with a consequential negative effect on growth in the infant (54). To achieve adequate growth, the protein to energy ratio should be maintained between 7.5% and 12%. This could be achieved by using a higher energy infant formula (which also has a higher protein content), or concentrating a standard infant formula along with a glucose polymer for term infants. Fortifier may be added to expressed breast milk or a formula for preterm infants can be used if breast milk is not available.

Where infants are managed outside of CHI specialist centres, the addition of a glucose polymer may be more challenging as appropriate scoops or specialist formula may not be readily available. In the absence of a specialized CHI dietitian, a paediatric tertiary centre dietitian or neonatal dietitian should be contacted to provide appropriate support.

#### Breastfeeding

If a mother wishes to breastfeed, support should be provided to express regularly to protect her milk supply, and to allow her breastmilk to be given as expressed breast milk (EBM). The duration of EBM use is expected to be moderately short and likely to be dependent on diazoxide dose, response and individual circumstances. If fluid intake is restricted, it may still be possible to put the infant to the breast for short periods which can help with bonding and maintain milk supply but requires agreement with the treatment team. Advice should be sought from specialist staff in this area such as breastfeeding advisors to increase the likelihood of success. Additional carbohydrate in the form of glucose polymers can be added to EBM if blood glucose levels are not maintained by medication alone.

Breast milk is naturally low in protein; the use of breastmilk fortifiers for pre-term infants should be considered over the simple addition of glucose polymers to ensure maintenance of the protein to energy ratio. Breast milk fortifiers are not prescribable in the community and therefore, if needed a regular hospital supply following discharge home may be required. This will need to be agreed with the wider community team. Alternatively, a protein supplement such as Protifar™ may be used to ensure adequate protein intake.

#### Continuous feeding

Some infants require feeding at least every 3 hours, if not more regularly, both day and night to ensure glycaemic stability. However, this can become exhausting and unsustainable for the parents. In such cases continuous feeds through a nasogastric or gastrostomy tube may be required.

If an infant is to be discharged home on pump feeding, a gastrostomy tube is recommended over a nasogastric tube to prevent aspiration of the feed. Additional advice on safety should be provided to the parents, e.g., how to prevent the risk of the tube becoming entangled around the neck at night. It may be important for parents to be in proximity to the child at night to hear the pump alarm in the event of a pump occlusion. To mitigate the risk of pump failure, a real-time continuous glucose monitor (CGM), with an alarm to detect hypoglycaemia, could be considered. Additionally, in the event of pump failure and subsequent risk of hypooglycaemia, the patient must be provided with a spare feeding pump to ensure that continuous feeding can be maintained.

#### Slow release carbohydrates

Older children may require additional glycaemic stability through the addition of slow release carbohydrates such as uncooked corn starch (55). Such supplements are preferably used in children older than 2 years with intestinal disaccharidases capable of breaking down carbohydrate in the form of starch. Corn starch, mixed in fluids at room temperature, may be commenced at 0.5g/kg/dose and increased gradually to a maximum of 2g/kg/dose to optimize therapeutic benefit while preventing side effects such as abdominal pain and loose stools. Corn starch is generally given with a pre-bedtime feed or snack but can be used up to 3-4 times a day if indicated and tolerated.

#### Weaning

Introduction to solid foods should be commenced when the infant displays normal feeding cues, has good head control, and be no later than 6 months for term infants. For preterm infants, the local neonatal team/dietitian should be consulted for advice on progression to solid foods. Normal healthy weaning advice is recommended using complex carbohydrate foods with avoidance of simple sugars in the early stages until the infant is on a mixed diet. High sugar purees are absorbed rapidly with resultant hypoglycaemia but with potential for post prandial hypoglyaemia and therefore best avoided, except for the treatment of acute hypoglycaemia.

#### Growth monitoring

Careful monitoring of weight and overall growth should be a priority for all patients so that feeds can be adjusted to optimize growth. For infants on diazoxide, fluid volumes are restricted with the potential to constrain growth. Thus, dietetic overview is essential through liaison with community staff e.g., health visitors to ensure that the infant receives appropriate nutritional advice. Regular weighing will ensure that feed volumes can be adjusted according to weight change to optimise growth.

Clear written instructions should be given when additional glucose polymers are added to feeds to ensure that the correct percentage is chosen. In some instances, weighing feed ingredients with scales, rather than the use of scoops, may be preferable for simplicity of formulation and accuracy of constituents (56).

#### Feeding problems

A proportion of patients with CHI have significant challenges with oral feeding (57, 58) requiring support from a Speech and Language Therapist (SLT) at an early stage. Feeding problems can occur early precluding establishment of milk feeding and successful progression with weaning and textures. Long term tube feeding can impair oral feeding progress with the development of oral aversion. The exact cause of feeding problems is not known but is most likely multifactorial, with complex entangled interplay of therapeutic, environmental, developmental and psychosocial issues (16, 59). The management of feeding problems in CHI requires early recognition and timely multidisciplinary involvement to mitigate such factors.

The use of anti-reflux medication such as proton pump inhibitors and thickened feeds may reduce vomiting and therefore address a few components in the range of causative factors associated with feeding problems. Parents should receive consistent support from SLT to allay frustration and feed related anxiety. SLT should also aim to support other caregivers, including nursing staff who often actively feed infants in the hospital and raise awareness of cue-based feeding practices to minimise the risk of worsening of feeding problems. While feeding problems are common in CHI patients, these should not preclude or dissuade oral feeding. The treatment team incorporating a SLT, should have a proactive aim to allow at least partial oral feeding, appropriate to the child’s age and developmental level. This remains relevant when beginning weaning and progressing through textures. At all stages in managing feeding difficulties, it is important to be responsive to individual acceptance of oral intake. Pushing oral feeding at an early stage in the presence of stress cues will adversely result in later food refusal that might be more challenging.

In some infants, feeding problems are complicated by intolerance to cow’s milk protein which may improve by changing to a more appropriate feed. If changing feeds, glucose levels should be monitored to ensure clinical stability during the transition period.

#### Intercurrent illness/surgery advice

Those infants who are on medication and/or additional carbohydrates should be provided with an intercurrent illness plan (emergency regime) which includes instructions on the use of a glucose polymer at a percentage suitable for the age of the child (**Appendices 2, 3**). Parents should be given written instructions on when and how to use emergency regimens. Adequate supplies must be maintained, especially for periods when they are away from the home such as during holidays. The emergency feeds should be tried when the child is well to ensure it will be taken when needed during illness episodes. Glucose polymer drinks can be flavoured with squash or cordial, if preferred, to improve palatability. During severe diarrhoeal/vomitting illnesses medical advice should be sought and hospital admission may need to be considered. In all cases of supplementation with glucose polymers, good oral hygiene should be maintained and regular dental review advised to minimize the potential for dental caries from excess sugar intake. Prolonged periods of fasting should be avoided, including those for procedures needing general anaesthesia. Administration of intravenous fluids with glucose with regular monitoring during the procedure is recommended to avoid hypoglycaemia. If needed, advice should be sought from the specialist centre.

#### Blood glucose monitoring

The ongoing monitoring of children with CHI in hospital primarily involves regular point of care (fingerprick age >1yr or heelprick age <1yr) glucose testing. The number of tests per day will depend on individual circumstances including response to medications, frequency of feeds and severity of illness. For patients with unstable glucose levels on a high intensity ward such as neonatal intensive care unit, blood glucose is typically checked 1-2 hourly until stability is achieved. Following stabilisation, most patients require 2-4 hourly blood glucose testing prior to feeds.

Point of care testing is accurate and laboratory glucose testing will not be routinely required. At discharge, patients should receive training in the use of a standard hand held blood glucose meter. Such meters are less accurate than point of care testing devices but are nonetheless widely used as home monitoring devices.

**Figure 3:**
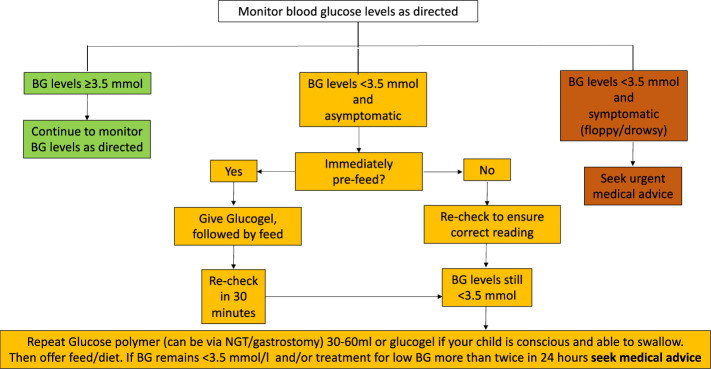
Flowchart for blood glucose monitoring for families. BG, blood glucose.

Blood glucose monitoring frequency at home is guided by the severity of illness, propensity to hypoglycaemia and need for adjustment to medications. For instance, when diazoxide dosage is actively reduced, a period of frequent monitoring may be required to reiterate glycaemic stability. There is no universal agreement on what constitutes glycaemic stability; however, by consensus, hypoglycaemic episodes (glucose < 3.5 mmol/L) exceeding two per week would imply imperfect control. For any hypolycaemia at home, parents are advised to follow local hypoglycaemia algorithms. An example of such an algorithm is provided in **Figure 3**.

### Continuous glucose monitoring

#### CGM in the hospital setting

For most patients, intermittent frequent blood glucose testing provides adequate information for monitoring in hospital. Continuous glucose monitoring (CGM) may be additionally helpful, owing to high frequency sampling providing an almost continuous profile. However, unlike diabetes, evidence for use in CHI is scant (60–62). Accuracy is lower than that of glucometer testing and any hypoglycaemia detected by a CGM device must be verified with a point of care glucometer before treatment is administered (62, 63). In hospital, CGM can be used to provide reassurance about normoglycaemia and thus reduce the number of point of care glucose tests (64). CGM offers additional utility in guiding point of care tests to times of possible hypoglycaemia in between routine tests, a feature that is beneficial in unstable patients.

#### CGM in the outpatient setting

CGM is not routinely used for home monitoring of CHI and is not supported by nationally agreed funding schemes (outwith individual funding requests). Thus, provision of CGM to patients in the outpatient setting is ascertained on a case by case basis using unpredictable streams of funding (65, 66).

The advantages and disadvantages of CGM in the hospital setting apply equally to the outpatient setting. However, the problems with inaccuracies of current CGM devices are amplified in the outpatient setting detracting from widespread use (66). There is, as yet, no published evidence of the utility of CGM as a standalone tool in reducing hypoglycaemia in CHI (67). CGM at home should be reserved for pattern recognition rather than for the acute detection of hypoglycaemic episodes. Parents should be counselled that CGM does not replace home blood glucose testing and that missed hypoglycamia remains a possibility due to inaccuracy in the present generation of CGM sensors.

### Investigations in CHI; role of genetics

#### Aetiology

Genetic causes for CHI can be found in 45-80% of cases, with the pick-up rate influenced by the screening methods employed by the genetics laboratory (68). The majority of pathogenic variants (69, 70) affect the ATP-sensitive K^+^ (KATP) channels in the pancreatic beta-cells, whilst others affect the intracellular proteins and enzymes involved in regulation of the insulin secretion pathway. Patients with a known genetic cause have variable disease trajectories with some achieving remission; however, recessively-acting variants in the KATP channel genes are more likely to be persistent (9) although with the potential for reduced disease severity with time (71). Similarly, those who remain without a genetic diagnosis following testing are more likely to achieve disease resolution with time, although disease trajectory is inconsistent and duration is unpredictable (72, 73).

CHI can be associated with other metabolic and genetic syndromic conditions such as Turner syndrome, congenital disorders of glycosylation and Beckwith-Wiedemann Syndrome (9, 11, 74, 75). Healthcare professionals managing patients with CHI are therefore advised to consider syndromic diagnoses and seek a genetic review if suspicion is aroused.

Disease-causing variants in the *ABCC8* and *KCJN11* genes which encode the KATP channel, can be inherited by both autosomal dominant and recessive routes. Dominantly inherited variants typically cause diffuse CHI, while recessively inherited variants cause both diffuse and focal CHI. The latter occur with paternal inheritance of a recessively acting KATP channel variant and concomitant loss of the maternal chromosomal 11p15 region within the pancreatic tissue. As a consequence, a focal lesion develops through clonal expansion of mutated β-cells which are dysregulated and secrete excess insulin. By contrast, recessively inherited biallelic variants invariably cause diffuse CHI with typical pancreatic histopathology (76).

Although an exhaustive list of the genetic causes of congenital hyperinsulinism has not been provided in this consensus, a few relatively common causes have been noted. Pathogenic variants in the gene encoding glutamate dehydrogenase (*GLUD1*), are associated with hyperinsulinaemia/hyperammonaemia syndrome where patients develop relatively mild hyperammonaemia (2-3 times the upper limit for serum ammonia) and a post-prandial hypoglycaemia aggravated by protein intake (11, 77, 78). *GLUD1* variants are dominantly acting but are most commonly sporadic in origin. These patients may have seizures which are unrelated to the severity of the hypoglycaemia and may require a protein restricted diet to manage hypoglycaemia (74, 77–79).

Another dominantly inherited cause of hyperinsulinism is due to activating variants in the gene encoding glucokinase (*GCK*). Patients with activating variants in *GCK* may present well beyond infancy and have variable expression in the same family. Recently, variants in a non-coding region of hexokinase 1 (*HK1*) that disrupts a regulatory element controlling gene expression have been described in patients presenting with CHI. Patients show a variable phenotype ranging between transient CHI and severe CHI requiring subtotal pancreatectomy (80).

Genetic investigations should be widened to include variants associated with both CHI and other conditions such as hypopituitarism, as in those with *FOXA2* variants (81). Such genetic testing is best undertaken by targeted gene panel testing where rapid turnaround times are not essential.

The genetic diagnosis of CHI should be confirmed by specialist genomic laboratories with appropriate technological expertise and experience. Accurate genotyping is important for clinical decision making (**Figure 2**) for optimal outcomes of CHI and for the prevention of unnecessary and harmful investigations that are not required in all cases. For a wider discussion of genetic aetiology and an up to date discussion of laboratory testing practices to investigate underlying cause, comprehensive open access reviews may need to be consulted (68).

#### Genetic testing strategies

Genetic testing in CHI patients should be undertaken at an early phase of clinical management and guided by the response to first line treatment with diazoxide (9, 11). Rapid *ABCC8*/*KCNJ11* sequencing is required in severe cases with high GIR and medical instability. Genetic testing should also be considered in those with persistent CHI (> 6 months by consensus) requiring diazoxide > 7mg/kg/day as they may have pathogenic variants in non-KATP channel genes (9). In such patients, gene panel testing should be considered as a less expensive alternative with greater coverage of CHI associated genes, although with a longer reporting turnaround time (68). In those with persistent CHI but diazoxide < 7 mg/kg/day, genetic testing may also be considered if there is no evidence for gradual dose reduction over time. In patients with CHI due to perinatal stress induced hyperinsuinism, genetic testing is not routinely recommended as CHI is unlikely to be of genetic aetiology and very likely to remit (82). It is however important to consider genetic testing, where there is a family history of early diabetes, even if diazoxide responsive, as this may be due to *HNF1A*, *HNF4A* variants (83).

#### Imaging

18-fluoro-dopa PET imaging with CT or MR modalities is indicated for the detection and localisation of focal lesions (84). As described above, a focal lesion is likely with the detection of a previously reported paternally inherited recessive variant in *ABCC8/KCNJ11*. In such cases, the CHI centre should be contacted for a discussion regarding the requirement, urgency and process for organising a scan. In case of novel variants in *ABCC8/KCNJ11* with no prior information regarding recessive/dominant inheritance, an 18-fluoro-dopa PET-CT/MR scan is also indicated. In this scenario (and in those without gene variants) the need for this scan should be reviewed in the context of illness severity. Patients with stable glycaemic profiles on low to modest doses of diazoxide (i.e. < 10 mg/kg/day) are unlikely to have focal CHI due to KATP channel disruption; in such cases, the decision for 18-fluoro-dopa PET-CT/MR scanning should be a lower priority, unless progressively increasing doses are required to maintain glycaemic stability. It is now recognised that variants in KATP channel genes may be limited to the pancreas and not be present in blood lymphocytes (85). In the absence of a known genetic aetiology from peripheral blood sampling, the presence of persistent hypoglycaemia due to CHI and ongoing need for therapy beyond age 2 years (by consensus) merits the consideration for an 18-fluoro-dopa PET-CT/MR scan (**Figure 2**).

It is recognised that PET imaging is the best available investigation to localise a focal lesion. However, scan findings are not always concordant with histological outcomes; therefore, treatment teams should be alert to the possibility of potential mislocalisation or a missed lesion on imaging. If a scan outcome is uncertain there is no evidence to suggest a repeat scan, unless the initial scan was of uncertain quality. Alternative radiotracers such as gallium-exendin have been developed (86), but it is unclear if efficacy overrides that of standard 18-fluoro-dopa PET imaging which has been shown to be highly sensitive (>95%) in the detection of focal lesions (87).

#### Surgery

In patients with a likely diagnosis of focal CHI (following diagnostic genetics and 18 fluoro-dopa PET-CT/MR scanning (88)), focal lesionectomy at a CHI specialist centre is the treatment of choice. Laparascopic lesionectomy is recommended for removal of lesions in the body or tail of the pancreas, as recovery times are short. However, the choice for minimally invasive laparacopic versus open laparatomy is predicated on individual circumstances and preference of the surgeon. In contrast to focal CHI, subtotal pancreatectomy is considered as a last resort in diffuse CHI not responsive to medical therapy. In all cases, the surgical team should be assisted by the CHI medical team to manage intraoperative and post-operative hypoglycaemia, hyperglycaemia and associated medical conditions. Additionally, frozen section histopathology is advised for all surgical resections, most importantly in focal lesionectomy, to identify and resect the focal lesions (89).

#### Discharge from hospital

Prior to discharge the infant should undergo a safety fast to ensure a reasonable fasting interval at home. The duration of this fast is intended to replicate a reasonable overnight food free interval that fits into parental routines of care. An age-appropriate safety fast should be performed in all patients with a diagnosis of CHI, including those infants managed with additional CHO alone and those with transient CHI. The safety fast should be considered when the infant feeds at 3 hourly intervals and ideally at 4 hourly intervals with stable glucose levels. The duration of the fast can vary between 6 and 8 hours, depending on the age of the child and expected food free interval, although longer fasts may be preferred by other centres to review capacity for ketogenesis. In contrast to prolonged fasts (favoured by centres outside the UK) that deviate from physiological fasting intervals, a shorter fasting period has greater practical application and is accepted by consensus to convey safety reassurance to families.

During the safety fast, blood glucose should be checked pre feed and then hourly from the 4^th^ hour after the last feed. Absence of end of fast hypoglycaemia (glucose < 3.5mmol/L) is considered a satisfactory outcome. If at any time during the fast, blood glucose dips below 3.5mmol/L, the fast should be discontinued and the patient fed immediately. At this stage the treatment team should review medication and feeds and consider a repeat fast after suitable adjustments. At discharge, an individualised care plan based on local need and family preferences should be discussed and a copy given to the parents. An example of such a plan is provided in **Appendix 4**.

#### The role of the extended multidisciplinary team

As CHI is a complex and challenging disorder, an MDT approach is strongly recommended. The MDT composition will depend on local availability of resources but should include paediatric endocrinologists (or specialists in inherited errors of metabolism), specialist nurses and dietitians. The MDT may also include, variably, clinical psychologists, SLT, surgeons, radiologists, nuclear medicine physicians and histopathologists. Specialist nurses have an important role in aiding the understanding for the family, providing them with emotional support during the hospital stay and arranging follow-up at discharge. They are a valuable resource for ward staff managing patients with CHI, providing training, education and expertise in optimising treatment outcomes. The specialist CHI nurse may also assist by providing nurse led outpatient follow up appointments, often through virtual consultations, thereby providing much needed support for parents and families.

The role of the MDT is relevant in long term follow up when neurodevelopmental outcomes and progress through school become more apparent. At all stages, MDT support is helpful for active guidance of treatment and ancillary issues not only for the parents, but also for other professionals around the child, for instance in school. An example of a school plan is provided in **Appendix 5**.

#### Long term Management

CHI patients should be monitored in the outpatient clinic 3-6 monthly, to assess growth, development, and responsiveness to treatment. More frequent review, often in virtual consultations, may be required to optimise therapy. If treatment is minimal, e.g. requirement for diazoxide <2-3mg/kg/day or octreotide <3mcg/kg/day with satisfactory glucose profiles, a trial of treatment withdrawal to assess disease remission should be considered. Consensus criteria for ascertaining remission include tolerance of an age-appropriate safety fast with suppressed insulin and robust ketone production (typically >1.5 mmol/L) by laboratory analysis at the end of the fast (71). A satisfactory profile on frequent home glucose monitoring may also suffice in patients with lesser duration and severity of illness, precluding the necessity for hospital admission and a rigorous fast.

Spontaneous resolution is expected in transient CHI related to perinatal stress. The probability of resolution is positively correlated with the absence of gene variants and a satisfactory response to low dose diazoxide. CHI due to pathogenic variants in *HNF4A* is often transient and some cases with dominantly acting *ABCC8/KCNJ11* may also resolve, but do so variably (90–92). Some infants with *HNF4A*, *HNF1A* and dominantly acting *ABCC8* variants may develop diabetes later in life, and therefore require advice and monitoring (83, 93) Some children develop idiopathic ketotic hypoglycaemia following resolution of CHI (94, 95) while in others, hypoglycaemia may recur. Therefore, except for perinatal stress related CHI, a follow up plan, even if infrequent, is recommended. Parents are advised to monitor glucose at home during intercurrent illness episodes, particularly if reluctant to have food or drink by mouth.

#### Neurodevelopmental outcomes

Abnormal neurodevelopmental outcomes are present in a significant number of children with both persistent and transient forms of CHI (96, 97) with frequency up to 48% in those with severe forms. While it is important to prevent neuroglycopaenia, it is also important to assess developmental progress in follow up. A developmental follow up schedule alongside medical review will enable screening for early problems, thereby facilitating early referral to community services for rapid intervention. Screening for neurodevelopmental outcomes could be undertaken from age one year (**Appendix 6**), although evolving abnormalities may present later and ongoing neurodevelopmental assessment should be considered, especially with parental concerns (98). In older children, and in those with screening deficits, standardised assessment of cognitive function may be required. Brain imaging is not usually part of standard clinical review as anatomical abnormalities do not necessarily correlate with functional outcomes. However, in those with severe neuroglycopaenia, visual cortical function loss or recurrent seizures, brain imaging may be helpful for review by paediatric neurologists.

#### Post pancreatectomy diabetes

Following sub-total pancreactomy, diabetes is common, with some cases developing diabetes shortly after surgery and some many years later. In the majority, diabetes develops within 10 years after surgery (73, 99, 100). Monitoring of pancreatic exocrine function is also required following sub-total pancreactomy. Faecal elastase should be measured annually. Pancreatic enzyme replacement therapy such as Creon®, should be considered, although not all patients will be symptomatic despite a low faecal elastase level (100).

#### Future therapies

Standard therapies are currently limited to a choice of diazoxide and octreotide and are often complicated by side effects (9). This has prompted the use of alternative therapies in observational studies such as sirolimus (101). However, sirolimus can be associated with side effects and should be used with caution in exceptional circumstances (102–104). The use of nifedipine, a calcium channel blocker, is not generally advocated for use (105).

New therapies are currently in clinical trials; these include soluble glucagon analogues (26, 106), long-acting glucagon, somatostatin receptor type 5 (SSTR5) agonist, monoclonal allosteric antibodies targeting the insulin receptor (107) and glucagon-like peptide-1 receptor (GLP-1r) antagonists. However, these are not yet available for routine clinical use.

#### Parent perspectives

**Figure 4:**
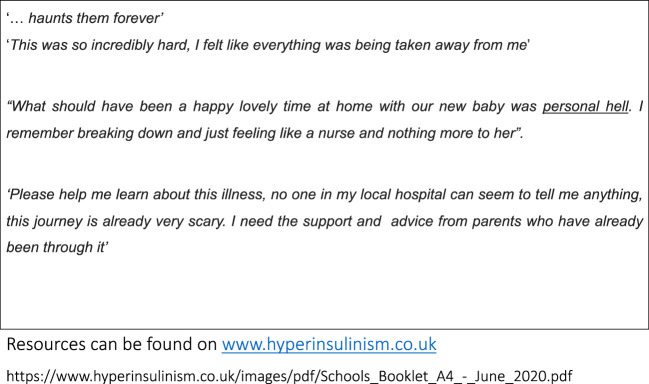
Quotes from parents describing their feelings in living with children with CHI.

The Children’s Hyperinsulinism Charity (CHC) is the registered patient charity for CHI in the UK. They offer support for parents and families living with CHI and interact and collaborate with clinical teams and organisations involved in healthcare provision for CHI patients. Useful information and resources can also be found on their website. (www.hyperinsulinism.co.uk), which is very helpful for parents especially during the initial diagnosis. As an important patient voice, they represent the perceptions of the community, represented as quotes ad verbatim in **Figure 4**. These quotes represent feelings resulting from a combination of stress and anxiety at diagnosis, prolonged hospital stay, inadequate bonding, sleep deprivation, fear of alarms, information gap, preparing medications, feeding problems, monitoring requirements, uncertain prognosis and many other factors. Parents often have to come to terms with a potential long-term illness and disability with strenuous feeding and sleep schedules in their child, They also have to live with with long-term caring responsibilities and realise that the burden of care for a child with CHI is much greater than another child of the same age, thereby adversely affecting the quality of family life. Treatment teams should recognise the strength of such feelings and work closely with organisations like the CHC as an important component in the networked care of CHI.

## Conclusions

CHI is a complex and difficult disease that usually presents in infancy but has long term effects resulting in multiple problems manifesting throughout childhood and later life. A UK wide consensus of healthcare professionals and patient representatives involved and experienced in the care of CHI patients has synthesised current evidence, practical considerations and harmonised practices to provide narratives and recommendations outlining a standardised approach to patient care with emphasis on partnership and collaboration involving treatment teams, parents, young people and relevant organisations.

## Author contributions

MGS devised this project and led on writing and coordination. RC and PC contributed to drafting and review of section on surgery. All authors contributed to the article and approved the submitted version.
