# Supplementary material for: Standardised practices in the networked management of congenital hyperinsulinism: a UK national collaborative consensus
Source: Front Endocrinol (Lausanne). 2023 Oct 30;14:1231043. doi: 10.3389/fendo.2023.1231043 (PMC10646160; doi:10.3389/fendo.2023.1231043)
Supplement: Supplementary file 1 [file DataSheet_1.docx]

**APPENDIX 1**

**Infant milks and supplements**

***Important notes:***

1. *The values in this table are correct as of April 2023 but can change over time so accuracy should be checked with the manufacturer.*
2. *The range of feeds provided here are not an exhaustive list and other feeds are available. The aim of this table is to provide a quick starting point for feeds management*

| **Infant Milks**  ***Nutritional information should be checked with manufacturer in case of updates*** | **Energy**  **kcals / 100ml** | **Protein**  **g/ 100ml** | **CHO**  **g/ 100ml** | **Protein: Energy ratio - %** |
| --- | --- | --- | --- | --- |
| EBM  (These are average figures. Content may vary) | 69 | 1.3 | 7.2 | 7.5 |
| **Fortifier added to EBM** | | | | |
| EBM and Cow and Gate Nutriprem Human Milk Fortifier | 85 | 2.6 | 8.7 | 12.2 |
| EBM and SMA Gold Prem Breast Milk Fortifier | 86 | 2.74 | 8.5 | 12.7 |
| **Term formulas** | | | | |
| Aptamil First infant milk | 66 | 1.3 | 7.3 | 7.87 |
| Cow and Gate First infant milk | 66 | 1.3 | 7.5 | 7.87 |
| SMA Pro First infant milk | 67 | 1.2 | 7.4 | 7.16 |
| Kendamil Classic First infant milk | 66 | 1.3 | 7.2 | 7.87 |
| Hipp Organic First infant milk | 66 | 1.28 | 7.0 | 7.76 |
| **Preterm formulas** |  |  |  |  |
| Cow and Gate Nutriprem 1 | 80 | 2.7 | 8.4 | 13.5 |
| SMA Gold Prem 1 | 80 | 2.9 | 8.1 | 14.5 |
| **Nutrient Enriched Post Discharge formula** | | | | |
| Cow and Gate Nutriprem 2 | 72 | 2.0 | 7.2 | 11.1 |
| SMA Gold Prem 2 | 73 | 2.0 | 7.7 | 10.9 |
| **High Energy formula** | | | | |
| Infatrini | 100 | 2.6 | 10.2 | 10.4 |
| SMA High Energy | 100 | 2.6 | 10.0 | 10.4 |
| Similac High Energy | 100 | 2.6 | 10.1 | 10.4 |
| Infatrini Peptisorb | 100 | 2.6 | 10.2 | 10.4 |
| **Other Specialist formulas** | | | | |
| Aptamil Pepti Junior | 66 | 1.8 | 6.9 | 10.9 |
| Aptamil Pepti 1 | 66 | 1.6 | 7.1 | 9.7 |
| Neocate LCP | 67 | 1.8 | 7.2 | 10.7 |
| Nutramigen 1 with LGG | 68 | 1.9 | 7.4 | 11.2 |
| SMA Soya Infant formula | 67 | 1.8 | 6.8 | 10.7 |

**Scoop chart for glucose polymers**

The figures below represent the weights of glucose polymers provided using the commonly recommended ‘**small purple scoop**’ as compared with the larger size of scoop which is provided in some product tins.

|  | 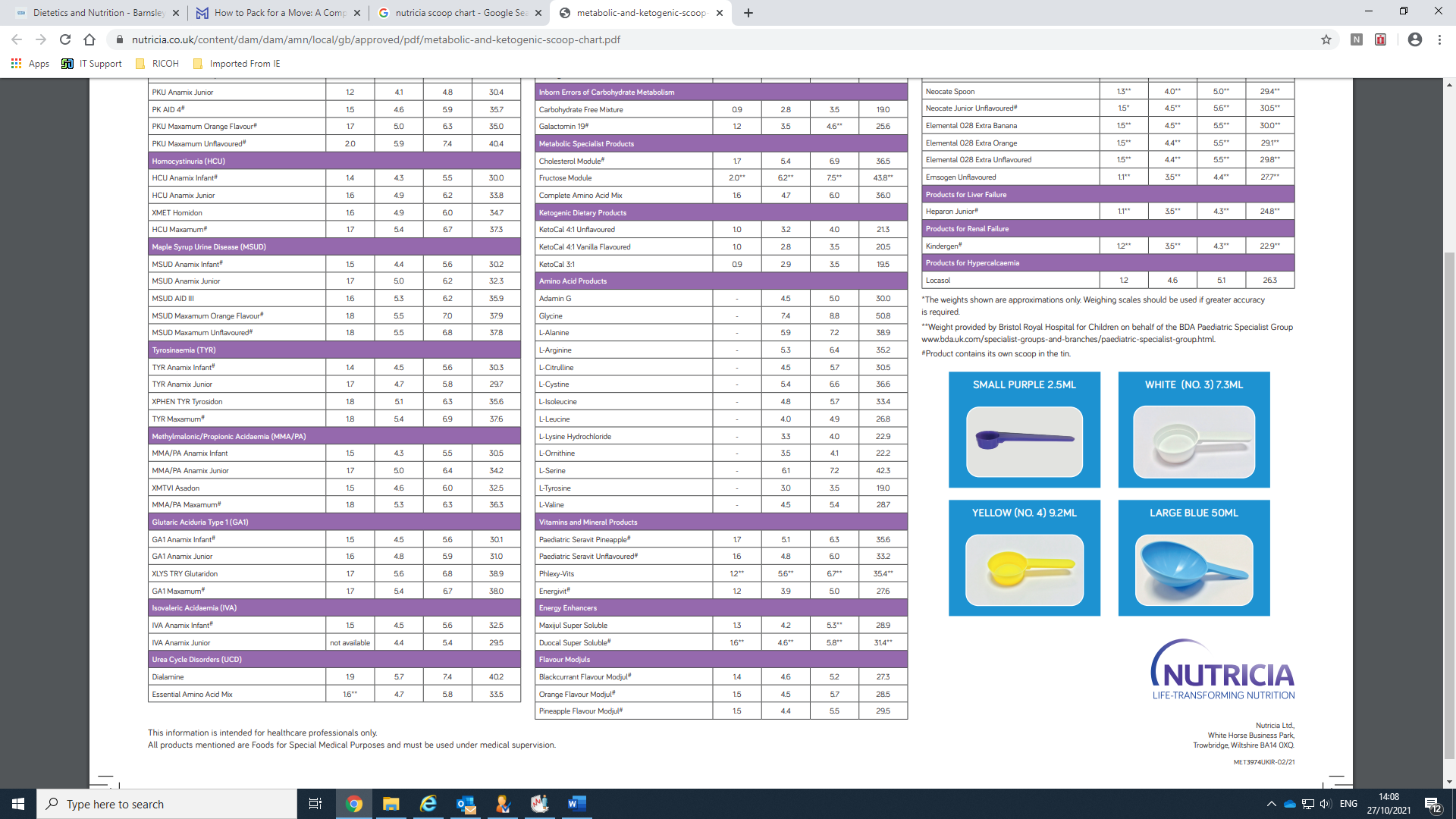**Small purple scoop (Nutricia/Instant Carobel)*** | **Weight of powder using products own scoop**  **(i.e. scoop provided in product tin)** |
| --- | --- | --- |
| **Vitajoule** | 1.5g | 12.3g |
| **Polycal** | 1.2g | 5.0g |
| **Maxijul** | 1.3g | No scoop provided |

*(Please note scoop weights of different products may vary over time - please contact your local paediatric/neonatal dietitian who can access to the most up to date information via the British Dietetic Association Paediatric Specialist Group Website: https://www.bda.uk.com/uploads/assets/7cd884f8-8f5f-48e1-ab8fb3d49a9b3c80/Scoop-Chart-Sep-2022-amended.pdf).*

**This scoop is available from Nutricia and is also found in boxes of Instant Carobel (which is manufactured by Nutricia)*

**Correct use of scoop**

Wash hands then fill the scoop as follows:

1. Dip scoop into the tin of powder

2. Lift heaped scoop from the tin without compressing powder against the wall of the tin

3.Level the scoop in the same way infant formula powder should be measured

**Percentage addition of carbohydrate**

- The percentage calculation will depend on which scoop is being used, which milk and what volume it is being added to.
- To work out percentage of any addition use the following calculation.

**Grams of additional carbohydrate ÷ volume of milk in millilitres x 100= % of addition**

- Add this figure to the percentage already in the milk (ie g of carbohydrate per 100ml of milk which can be ascertained from the table above/manufacturer)

*E.g. 1 small purple scoop of Vitajoule in 60ml of Nutriprem 1 formula*

*1.5g ÷ 60ml x 100= 2.5% of additional carbohydrate*

*Therefore 2.5% additional carbohydrate + 8.4% carbohydrate from Nutriprem 1 formula = 10.9% total carbohydrate*

- Where immediate access to specialist dietitian/ feed kitchen is not possible, an alternative would be to add 1 scoop of Vitajoule  (Nutricia/Carobel-1.5g/scoop) to 50mls of milk which will give an additional 3% of carbohydrate

**Examples of infant milks made up with addition of glucose polymer**

| **Infant Milks with addition of glucose polymer** | **Energy**  **kcals / 100ml** | **Protein**  **g/ 100ml** | **CHO**  **g/ 100ml** | **Protein: Energy ratio - %** |
| --- | --- | --- | --- | --- |
| Aptamil First infant milk + 3% glucose polymer | 78 | 1.3 | 10.3 | 6.7 |
| SMA Gold Prem 1 + 3% glucose polymer | 92 | 2.7 | 11.1 | 11.7 |
| Cow and Gate Nutriprem 2 + 5% glucose polymer | 92 | 2.0 | 12.2 | 8.7 |
