# Supplementary material for: Standardised practices in the networked management of congenital hyperinsulinism: a UK national collaborative consensus
Source: Front Endocrinol (Lausanne). 2023 Oct 30;14:1231043. doi: 10.3389/fendo.2023.1231043 (PMC10646160; doi:10.3389/fendo.2023.1231043)
Supplement: Supplementary file 2 [file DataSheet_2.docx]

***Appendix 2***

**Information for Patients/Parents**

***Emergency plan for Congenital Hyperinsulinism***

***For under 1 year of age***

**Reason for an emergency plan**

As part of their Congenital Hyperinsulinism (CHI) management your baby will be on set volumes of feeds at set time intervals. If they are unwell and not able to take their feed they will be at higher risk of their blood glucose level dropping. For this reason we give an emergency plan to our babies with CHI to use during times of illness.

This emergency plan uses a glucose polymer (Vitajoule; Super Soluble Maxijul; Polycal) to provide your baby with enough carbohydrate to maintain their blood glucose if they are unwell and refusing their feeds or have vomiting/diarrhoea. You may already be using a glucose polymer in your baby’s usual feeds.

This emergency plan should only be used during times of illness or when your baby is refusing feeds. It should not be used as part of their daily blood glucose management.

**This plan should not be used to treat hypoglycaemia. If hypoglycaemia occurs whilst using the emergency plan you should still use your hypo treatment as outlined by your CHI nurse.**

**Instructions on how to use this document**

- Ensure you have your glucose polymer from your hospital or GP. It will be available on repeat prescription.
- Identify your child’s emergency plan recipe and drink size based on their current age (use table on page 3).
- Make up the drink as per the recipe.
- Follow the flow chart.
- Once your tub of glucose polymer is open put in a request with your GP for another one so that you are never caught short. Once open it will last for 1 month if it is stored in a cool dry place.

**Under 6 months**

It is important to use cooled boiled water to reduce your baby’s risk of exposure to bacteria that may be in the water.

**Guidelines for feeding your child during illness**

**Step 1: Possibly unwell or refusing feeds**

If you are worried about your baby because they appear unwell, vomiting or may be ill with a virus e.g. a cold, or if refusing food then **give a one off drink** using your emergency plan recipe

**Review in next 2 hours**

If illness improves and your baby is eating and drinking/ feeding normally then discontinue emergency plan

If illness continues move on to **step 2** below

**Step 2: Unwell and/or refusing meals**

Give emergency drink **every 2 hours during the day and every 3 hours at night** giving a total of 10 feeds in 24 hours.

**Is your child tolerating their emergency plan?**

**No Yes**

If your baby is able to drink their emergency drink and keep it down, continue to give it as directed above until they start to eat again. **Take your baby to their GP or walk in centre for assessment if this continues for more than 24 hours.**

If your baby is unwell, not feeding **and** unable to tolerate the emergency drink (not able to drink or vomiting it straight back) **you should take them to your local hospital** for a drip of 10% glucose (even if not hypoglycaemic).

**Step 3: Not tolerating emergency plan, attending hospital**

**Advice for doctors**:

- If hypoglycaemic and symptomatic give IV glucose 200mg/kg (2.5ml/kg10% glucose) followed by a continuous infusion of 5-10 mg/kg/min (3-6 ml/kg/hr) of 10% glucose + 0.45% NaCl or equivalent. Continue infusion until the blood sugar is stable and tolerating oral feeds.
- If asymptomatic or normoglycaemic but not tolerating oral feeds give the IV infusion without the initial bolus.

**Emergency plan recipe and how much to give**

The amount of emergency drink you give your baby will vary depending on your baby’s age. As your child gets older the amount you give them will increase to provide the appropriate amount of glucose for their age and size. Please see below for advice on how much emergency drink to give.

| **Age** | **Glucose Polymer recipe**  **(10% carbohydrate)** | **Amount**  every 2 hours in the day and 3 hours overnight |
| --- | --- | --- |
| 0-3 months | ***10% Carbohydrate***  10g of glucose polymer  add cooled boiled water up to 100ml | 70ml |
| 3-12months |  | 100ml |

Glucose polymer used:…………………………………………………

If using scoops – number of scoops to provide 10g Carbohydrate: …………

If you have to use the emergency plan – please let your endocrine nurse or dietitian know, so that further advice can be given.

Please show this document to your baby’s GP or any other doctor who may be seeing your baby.

Please contact the dietetic team or nurse specialist if you have any concerns about this protocol or your child’s treatment.

Dietitian:

Telephone:

Mobile:

Email:

Paediatric Endocrine Clinical Nurse Specialist:

Endocrine nurse mobile:

Endocrine nurse email:
