# Supplementary material for: Standardised practices in the networked management of congenital hyperinsulinism: a UK national collaborative consensus
Source: Front Endocrinol (Lausanne). 2023 Oct 30;14:1231043. doi: 10.3389/fendo.2023.1231043 (PMC10646160; doi:10.3389/fendo.2023.1231043)
Supplement: Supplementary file 3 [file DataSheet_3.doc]

**Emergency plan for Congenital Hyperinsulinism**

For over 1 year of age

**Information for Patients/Parents**

**Reason for an emergency plan**

As part of their Congenital Hyperinsulinism (CHI) management, your child will be on a feeding plan of regular meals or feeds to maintain their blood glucose level on a daily basis. If they are unwell and not able to eat or take their feed they will be at higher risk of their blood glucose level dropping. For this reason emergency plans are given children with CHI to use during times of illness.

This emergency plan uses a glucose polymer (Vitajoule; Super Soluble Maxijul; Polycal) to provide your child with enough carbohydrate to maintain their blood glucose if they are unwell and refusing food, their feeds or have vomiting/diarrhoea.

This emergency plan should only be used during times of illness when your child is refusing meals or not tolerating feeds. It should not be used as part of their daily blood glucose management. You should inform your HI team if you have to use the emergency plan.

When your child is well it is important that they eat regular meals and snacks which include starchy carbohydrates e.g. cereals, bread, rice pasta, potato, milk. A bedtime snack and breakfast are particularly important.

**This plan should not be used to treat hypoglycaemia. If hypoglycaemia occurs whilst using the emergency plan you should still use your hypo treatment as outlined by your CHI nurse.**

**Instructions on how to use this document**

- Ensure you have a supply of your glucose polymer from your hospital or GP. It should be available on repeat prescription.
- Identify your child’s emergency plan recipe and drink size based on their current age (use table on page 3).
- Make up the drink as per the recipe.
- Follow the flow chart.
- Once your tub of glucose polymer has been opened, put in a request with your GP for another one so that you are never caught short. Once open it will last for 1 month if it is stored in a cool dry place.
- Ensure that you have sufficient glucose polymer to give to relatives or nurseries that your child goes to on a regular basis and a copy of the plan is given to staff and relatives
- You should let your CHI nurse or dietitian know if you use the emergency plan.

**Guidelines for feeding your child during illness**

**Step 1: Possibly unwell or refusing meals**

If you are worried about your child because they appear unwell, vomiting or may be ill with a virus e.g. a cold, or if refusing food then **give a one off drink** using your emergency plan recipe

**Review in next 2 hours**

If illness improves and your child is eating and drinking normally then discontinue emergency plan

If illness continues move on to **step 2** below

**Step 2: Unwell and/or refusing meals**

Give emergency drink **every 2 hours during the day and every 3 hours at night** giving a total of 10 feeds in 24 hours.

**Is your child tolerating their emergency plan?**

**No Yes**

If your child is able to drink their emergency drink and keep it down, continue to give it as directed above until they start to eat again. **Take your child to their GP or walk in centre for assessment if this continues for more than 48 hours.**

If your child is unwell, not eating **and** unable to tolerate the emergency drink (not able to drink or vomiting it straight back) **you should take them to your local hospital** for a drip of 10% glucose (even if not hypoglycaemic).

**Step 3: Not tolerating emergency plan, attending hospital**

**Advice for doctors**:

- If hypoglycaemic and symptomatic give IV glucose 200mg/kg (2ml/kg 10% glucose) followed by a continuous infusion of 5-10 mg/kg/min (3-6 ml/kg/hr) of 10% glucose + 0.45% NaCl or equivalent. Continue infusion until the blood sugar is stable and tolerating oral feeds.
- If asymptomatic or normoglycaemic but not tolerating oral feeds, give the IV infusion without the initial bolus.

**How much emergency plan recipe and how much to give**

**The amount of emergency drink you give your child will vary depending on your child’s age. As your child gets older the amount you give them will increase to provide the appropriate amount of glucose for their age and size. Please see below for advice on how much emergency drink to give.**

| **Age** | **Glucose polymer recipe**  **In water (weight in g)** | **Amount to give**  every 2 hours in the day and 3 hours overnight |
| --- | --- | --- |
| 12m-18m | **15% carbohydrate**  **30g glucose polymer**  **Made up to 200ml with water** | 100ml |
| 18m-2 years |  | 120ml |
| 2-3 years | **20% carbohydrate**  **40g glucose polymer**  **Made up to 200ml with water** | 120ml |
| 3-5 years |  | 140ml |
| 5-8 years |  | 160ml |
| 8-10 years |  | 180ml |
| 10-11 years | **25% carbohydrate**  **50g glucose polymer**  **Made up to 200ml with water** | 180ml |
| 11-14 years |  | 200ml |
| 14-16 years |  | 220ml |
| 16+ |  | 240ml |

Glucose polymer used: …………………………………………………………………….

If using scoops – number of scoops to provide 10g Carbohydrate: ……………………

The Glucose polymer used may vary between different hospitals and the recipe for the Glucose polymer will be dependent on the scoop size, please consult your dietitian.

Scoops for the particular glucose polymer used, will be provided by the dietitian

Please show this document to your child’s GP or any other doctor who may be seeing your child.

Please contact the dietetic team if you have any concerns about this protocol or your child’s treatment.

Dietitian:

Mobile:

Email:
