# Supplementary material for: Standardised practices in the networked management of congenital hyperinsulinism: a UK national collaborative consensus
Source: Front Endocrinol (Lausanne). 2023 Oct 30;14:1231043. doi: 10.3389/fendo.2023.1231043 (PMC10646160; doi:10.3389/fendo.2023.1231043)
Supplement: Supplementary file 4 [file DataSheet_4.docx]

**Appendix 4**

Print on trust headed paper

**Congenital Hyperinsulinism**

Affix Patient label

**Discharge Check List**

|  | CHECKLIST | Completed by | Date |
| --- | --- | --- | --- |
| 1. | Discussion with specialist CHI center regarding ongoing management |  |  |
| 2. | Normal blood glucose profile (> 3.5 mmol/l) for 24-48 hours on feeding regimen baby will be discharged on. |  |  |
| 3. | Tolerated 6-8 hours safety fast without any hypoglycaemia. |  |  |
| 4. | No clinical evidence of fluid retention and pulmonary hypertension on discharge if on diazoxide. |  |  |
| 5. | Parents to be trained to identify signs of fluid overload and understand the need to fluid restrict to 130-150mls/kg/day if on diazoxide |  |  |
| 6. | Parents have received a written hypoglycaemia plan and are trained to act accordingly. |  |  |
| 7 | Parents given written information on hyperinsulinism |  |  |
| 7. | Parents to be provided with a glucometer and trained to check blood glucose levels |  |  |
| 8. | Parents should be advised to check pre-feed blood glucose levels at home for at least 1 week after being discharged. |  |  |
| 9. | Home supplies arranged as per local policy (blood sugar monitor, lancets, strips, medication and feeds) |  |  |
|  | Child registered with GP |  |  |
| 10. | Parents should have contact details of treating center including specialist nurse/dietetic team. |  |  |
| 11. | CHI team at Specialist Centre is informed about discharge so they can arrange follow up |  |  |
| 12. | Child should have open access to local hospital. |  |  |
| 13. | CHI team at Specialist Centre is informed about discharge so they can arrange follow up date |  |  |
| 14. | Follow up appointment arranged with general paediatrician at local hospital/shared care with regional paediatric endocrinologist |  |  |
| 15. | If applicable hypoglycaemia alerts added to patient notes |  |  |
| 16. | Where applicable, an ambulance alert should be added |  |  |
|  | Other |  |  |
|  | **Further information for families is available on CHI specialist website.** |  |  |
