# Supplementary material for: Standardised practices in the networked management of congenital hyperinsulinism: a UK national collaborative consensus
Source: Front Endocrinol (Lausanne). 2023 Oct 30;14:1231043. doi: 10.3389/fendo.2023.1231043 (PMC10646160; doi:10.3389/fendo.2023.1231043)
Supplement: Supplementary file 5 [file DataSheet_5.docx]

**APPENDIX 5**

**CHI Information for Schools**

Congenital hyperinsulinism (CHI) is characterized by inappropriate and unregulated insulin secretion from the beta-cells of the pancreas. In CHI, the beta-cells release insulin inappropriately all the time and insulin secretion is not regulated by the blood glucose level (as occurs normally). In CHI, too much insulin causes the blood glucose level to drop too low. High insulin levels prevent ketone bodies being made. This means that the brain is not only deprived of its most important fuel (glucose), but also ketone bodies which are used as alternative fuels. When the brain has no glucose or ketones to use as fuel then the child is at risk of seizures, loss of consciousness and even brain injury.

Treatment aims to keep a child’s blood glucose level stable between 3.5mmol/litre to 10mmol/litre. This can be managed by regular high carbohydrate feeds alongside medicines to reduce insulin secretion.  Sometimes the management of CHI can be complicated. However, once CHI is stable, a degree of normal life can be achieved. However, children and young people with CHI may still have issues with brain development causing memory and processing information so may need additional support. It is important to be aware of how hypoglycaemia can impact on learning and accessing the curriculum, causing attention, concentration, memory, and processing problems. Pupils may require extra support for information to be repeated and to help retain and record information, to ensure valuable teaching input has not been missed during hypoglycaemic episodes, or when leaving the classroom to manage hypoglycaemia, or attending hospital appointments.

A hypoglycaemic (hypo) episode will occur when blood glucose is less than 3.5mmol/litre. Some, but not all, children will notice they don’t feel right when their blood sugar levels start to go too low.

Common symptoms include:

- Feeling tired or sleepy
- Feeling wobbly or shaky
- Feeling dizzy
- Feeling hungry
- Feeling grumpy or angry
- Having a headache

If the teachers notice any of these signs, or the pupil is exhibiting unusual behaviour, they should check the pupil’s blood glucose level immediately and follow the emergency hypo plan below.

The pupil will have an individual hypo plan and will carry an emergency glucose product or snack with them at all times to deal with a hypoglycaemic episode. In an event of hypoglycaemia the teacher should follow the pupil’s emergency hypo plan plus inform the pupil’s parents.

An important part of treating CHI is to ensure that your pupil has regular and frequent snacks and drinks throughout the school day. These will be provided by the pupil’s family and should be carried with them at all times. The pupil should be allowed to leave lessons to have a snack or drink if needed.

The pupil will also need to check their blood glucose levels at regular intervals throughout the day. Children and young people are given a blood glucose monitor which measures the amount of glucose in the blood. This is done by a small finger prick that will give a single drop of blood to put on a strip. Advice regarding the timings of monitoring will be on an individual basis however pre - lunch and pre -  exercise is often suggested.  Some children will be wearing a device called a continuous glucose monitor and sensor.  This will assist in tracking the glucose levels and advice regarding this will be given on an individual basis. Additional checks may be needed if the pupil is unwell or takes part in energetic activities such as games and school trips.
