# Supplementary material for: Standardised practices in the networked management of congenital hyperinsulinism: a UK national collaborative consensus
Source: Front Endocrinol (Lausanne). 2023 Oct 30;14:1231043. doi: 10.3389/fendo.2023.1231043 (PMC10646160; doi:10.3389/fendo.2023.1231043)
Supplement: Supplementary file 6 [file DataSheet_6.docx]

**Appendix 6***- Developmental follow up schedule for first 2 years*

| **Timing** | **Type of Assessment** |
| --- | --- |
| 3-6 weeks | General Medical and developmental assessment |
| 6 months | General Medical and developmental assessment |
| 12 months | General Medical and developmental assessment +/- SOGS* |
| 18 months | Medical and developmental assessment +/- SOGS |
| 24 months | Medical and developmental assessment +/- SOGS and PARCA-R**; Vineland Adaptive Behaviour Scales Questionnaire 3^rd^ edition |

*SOGS- schedule of growing skills

**-PARCA-R- Parent Report of Children's Abilities-Revised
